# Accelerometer-Measured Physical Activity and Sedentary Time among Children in Japan before and during COVID-19: A Cross-Sectional and Longitudinal Analysis

**DOI:** 10.3390/ijerph20021130

**Published:** 2023-01-09

**Authors:** Chiaki Tanaka, Akiko Shikano, Natsuko Imai, Kar Hau Chong, Steven J. Howard, Kosuke Tanabe, Anthony D. Okely, Ellie K. Taylor, Shingo Noi

**Affiliations:** 1 Department of Human Nutrition, Tokyo Kasei Gakuin University, 22 Sanbancho, Chiyoda-ku, Tokyo 102-8341, Japan; 2 Faculty of Sport Science, Nippon Sport Science University, 7-1-1 Fukasawa, Setagata-ku, Tokyo 185-8508, Japan; 3 School of Health and Society and Early Start, Faculty of the Arts, Social Sciences and Humanities, University of Wollongong, Wollongong, NSW 2522, Australia; 4 School of Education and Early Start, Faculty of the Arts, Social Sciences and Humanities, University of Wollongong, Wollongong, NSW 2522, Australia; 5 Faculty of Humanities and Social Sciences, Teikyo Heisei University, 4-21-2, Nakano, Nakano-ku, Tokyo 164-8530, Japan

**Keywords:** accelerometer, physical activity, sedentary behavior, sleep, cognitive function, children

## Abstract

This study examined changes in physical activity (PA), sedentary behavior (SB), screen time, sleep, and executive function among Japanese preschoolers between COVID-19 pre-pandemic and pandemic periods, using cross-sectional and longitudinal data. Accelerometer data from 63 children aged 5–6 years were collected from three kindergartens in Tokyo, Japan, in late 2019 (pre-COVID-19). This was compared to the data of 49 children aged 5–6 years from the same kindergartens, collected in late 2020 (during COVID-19). Sixteen children in the pre-COVID-19 cohort also participated in the 2020 survey and provided data for the longitudinal analysis. The mean minutes of PA, SB, screen time, and sleep duration, as well as executive function, were compared between the pre- and during COVID-19 cohorts. After adjusting for school, sex, and accelerometer wear time, there were no significant differences in any of the measured outcomes between the two cohorts. However, the analysis of longitudinal data revealed significant increases in time spent in SB and on screens, and a decrease in light-intensity PA and sleep duration during the pandemic compared to the pre-pandemic period. Results suggest that, despite the COVID-19 pandemic, young children’s activity levels and SB did not significantly differ from pre-pandemic levels. However, school-aged children’s SB, light PA, and sleep time were affected, although this cannot be disentangled from the effects of the transition to school.

## 1. Introduction

The first case of coronavirus disease 2019 (COVID-19) in Japan was confirmed on 14 January 2020. In Japan, a state of emergency was declared across the country on 16 April 2020, but no lockdown was implemented. On 4 May 2020, the Ministry of Health, Labour and Welfare proposed a new lifestyle as “New Normal” in anticipation of lifting the state of emergency (which was lifted on 26 May 2020) [1]. On 22 May 2020, the Ministry of Education, Culture, Sports, Science and Technology (MEXT) published the “COVID-19 Infection Control Manuals and Guidelines for schools -New School Lifestyle” [2]. According to this document, students are instructed not to play in such a way that their bodies come into contact with each other, especially during recess periods, regardless of the levels of local infection [2]. With this, it is generally assumed that the school attendance rates and time spent in school decrease over this period, and that opportunities for physical activity (PA) also decreases during the school hours. 

Recently, Kharel et al. [3] conducted a systematic review to comprehensively review the findings from studies conducted in different settings and varying degrees of lockdown restrictions to inform policy decisions on the enforcement of lockdowns for subsequent waves of COVID-19 and future pandemics of a similar nature. Results showed that lockdown restrictions to curb the spread of COVID-19 mostly negatively affected children’s and adolescents’ movement behaviors worldwide. Children reported spending less time in PA and more time on screens during confinement relative to the period before the pandemic. However, children and adolescents who lived in areas with lower levels of restrictions, such as in Germany and Western Australia, were physically more active and used screens less than those who lived in areas with stricter lockdowns, such as in Spain. Children and adolescents also tended to sleep longer hours, with a later bedtime and wake-up time reported during the pandemic than before the pandemic. However, this review did not include studies conducted in Japan. As no lockdown was implemented in Japan, it is believed that the impact of COVID-19 may differ from that of other countries.

Previous systematic reviews have identified substantial evidence supporting the associations between the time spent in PA, sedentary behavior (SB), and sleep duration, both individually and in combinations, and a range of health and developmental outcomes in young children and school-aged children [4,5,6,7,8,9]. Recognizing the importance of an integrated movement approach, Australia and Canada have published their first 24-h movement guidelines (PA, SB, and sleep duration) for children aged under 5 years and 5–17 years [10,11,12,13]. In 2019, the World Health Organization (WHO) [14] also published global guidelines on 24-h movement behaviors for children aged under 5 years. In Japan, only 60%, 21.5%, and 68.7% of primary school children aged 6–12 years who live in urban areas meet the recommended daily durations of moderate- to vigorous-intensity PA (MVPA), SB, and sleep, respectively [15]. Among younger Japanese children, a previous study reported that 75.4%, 15.9%, and 68.1% of children aged 4.6 ± 0.2 years old met the PA, SB, and sleep duration guidelines, respectively [16]. Recently, Hyunshik et al. [17] reported that the compliance with 24-h movement guidelines significantly decreased during the COVID-19 pandemic compared to the pre-pandemic period among Japanese preschoolers (3.6 ± 0.3 years old). However, this study calculated the accelerometer-based MVPA time using the primary school children-specific conversion equations [18], which are likely to overestimate the metabolic equivalent results of the younger children [18]. Moreover, there is a lack of studies describing the impact of such behavioral changes on children’s executive function. 

This study aimed to examine the changes in objectively measured PA and SB, subjectively measured screen time and sleep, and directly assessed executive function of preschool-aged children during the COVID-19 pandemic in 2020 compared to the pre-pandemic period in 2019, using a repeated cross-sectional design. A longitudinal survey of changes in movement behaviors (PA, SB, screen time, and sleep duration) was also conducted by following a sub-sample of children in the 2019 survey who transitioned into primary schools in 2020. 

## 2. Materials and Methods

For the repeated cross-sectional study, participants were recruited using a leaflet, and all participants and their parents gave their written informed consents. The first round of data collection was conducted in October and November 2019 (pre-pandemic period), involving three kindergartens in the urban areas of Tokyo. In October and November 2020 (during the pandemic), we recruited a group of children from the same kindergartens again to provide data for the comparability analysis. For the longitudinal study, a sub-sample of children in the 2019 survey who had transitioned into primary schools in 2020 were invited to participate in the follow-up measurements (except cognitive function) in October and November 2020 using the same study protocol. The study protocol was approved by the Ethical Committee of the lead institution’s ethics committee (receipt number: 019-H081, approval date: 9 August 2019). 

Standardized data collection and cleaning procedures were followed across study phases to ensure data consistency and integrity [19]. Participants wore the ActiGraph GT3X+ (ActiGraph Inc., Pensacola, FL, USA) on their waist continuously for 7 days. Time spent in SB, light-intensity PA (LPA), and MVPA were calculated using Pate et al.’s cutpoints for data collected at pre-school age and Evenson et al.’s for data collected at primary school age [20,21]. We used the 20 min of consecutive zero counts’ criteria to identify and exclude non-wear time during the specified time period [22]. This included the time where the child removed the monitor for bathing, showering, and/or swimming. Accelerometer data were considered valid if it has at least 6 h of valid wear time per day for at least 4 days. 

Screen time (i.e., time spent viewing television and videos, playing electronic games, and using a personal computer) and nocturnal sleep schedule on a typical weekday and weekend day were reported by parents. The sleep duration was then calculated from the parent-reported bedtime and wake-up time, separately for weekdays and weekends. The average daily values of screen time and sleep duration at night were calculated by weighting for 5 weekdays and 2 weekend days, respectively. Parents were also asked to report their child’s participation in organized sports clubs. 

To measure the three fundamental constructs of executive function (i.e., inhibition, working memory, and cognitive flexibility), we used the iPad-based Early Years Toolbox (EYT). Inhibition was measured using the EYT Go/No-Go task, in which children resist a pre-potent impulse to tap the screen (‘catch the fish’) whenever an infrequent shark appears. Possible scores range from 0 to 1, with a higher score indicating better inhibitory control. Visual–spatial working memory was measured using the EYT Mr. Ant task, wherein children recall the spatial locations of colored ‘stickers’ placed on a cartoon ant. The number of stickers to be recalled is reflective of the working memory demand of the test item. Possible scores range from 0 to 8, with a higher score indicating better working memory. Cognitive flexibility was measured using the EYT Card Sorting task, wherein children must flexibly switch between rules for sorting stimulus (color, shape, or the rule being contingent on the presence of a border around the stimuli). Possible scores range from 0 to 12, with a higher score representing better cognitive flexibility. These measures have shown strong reliability and convergent validity with the existing measures, and developmental sensitivity [23]. In the longitudinal study, EYT could not be conducted in primary schools because headteachers of the schools did not want non-essential outside visitors to mitigate the risk of COVID-19 spread.

Body height and weight were measured, without shoes and with light clothing, to the nearest 0.1 cm and 0.1 kg, respectively. Body mass index (BMI) was calculated as weight (kg) divided by height (m) squared. Weight status was classified according to WHO growth reference for children and adolescents, and the national reference data for Japanese children (relative weight) [24,25].

For the repeated cross-sectional data, an analysis of covariance (ANCOVA) was applied to examine the differences in movement behavior variables between pre- and during-pandemic groups, with each movement behavior variable included as the dependent variable and the group (pre- and during-pandemic) as a fixed factor in the analyses. All analyses were further adjusted for sex (as a fixed factor), school (as a random factor), BMI, and accelerometer wear time (for PA and SB models only). The distributions of some variables were slightly skewed; therefore, the comparability analyses were repeated using the non-parametric test (Mann-Whitney). For longitudinal data, a paired sample t-test was used to compare baseline and follow-up measurements. The proportion of participants meeting the MVPA (accumulating 60 min or more of MVPA per day), screen time (no more than 2 h per day), and sleep duration (9 to 11 h of sleep per night) recommendations of the 24-hour Movement Guidelines for Children (5–12 years), both individually and in combination, were calculated [10,13]. Results are reported as mean and standard deviation (for continuous data) or frequency and percentage (for categorical data). Statistical analysis was performed using IBM SPSS statistics 23.0 for Windows (IBM Co., Tokyo, Japan). A *p* value of <0.05 was considered statistically significant.

## 3. Results

A total of 117 children were recruited in 2019; 54 were excluded from the current analysis due to the following reasons: withdrawal of consent (*n* = 3), invalid accelerometer data (*n* = 50), and bad physical condition during data collection (*n* = 1). This resulted in a sample size of 63 participants (36 boys and 27 girls) for the analysis of the 2019 survey (T1). Of this sample, 26 children had consented to participate in a follow-up survey conducted between October and November 2020 (T2), with 16 of them providing valid data for the longitudinal analysis. For the repeated cross-sectional survey in 2020, 128 children were recruited from the same kindergartens again. Seventy-nine children were excluded from the analysis due to withdrawal of consent (*n* = 1) and invalid accelerometer data (*n* = 78), leaving a sample size of 49 participants (23 boys and 26 girls) for the repeated cross-sectional analysis. 

The characteristics of study participants are presented in Table 1. For the repeated cross-sectional samples, the pandemic group, on average, had a significantly higher body weight and BMI than the pre-pandemic group. For the longitudinal sample, age, height, and body weight were significantly higher in the pandemic group compared to the pre-pandemic group, and there were no obese children in either of the groups. Table 2 shows the proportion of participants who met the MVPA, screen time, and sleep duration recommendations. The largest difference in the individual guideline adherence rate between the pre- and during-pandemic groups was observed for the SB recommendation.

For the repeated cross-sectional samples, the time spent at different PA intensity levels, SB, screen time, sleep duration, and executive function are shown in Table 3. There were no significant differences between the pre-pandemic group and the pandemic group for these variables. Similar results were obtained when the Mann-Whitney test was applied. However, the proportion of participants who reported participating in the organized sports clubs was larger among the pre-pandemic group (65.1%) than the during-pandemic group (22.4%). 

For the longitudinal sample, time spent at different activity intensity levels, SB, screen time, and sleep duration time are shown in Table 4. Compared to the measurements at T1, participants reported spending significantly more time in SB and on screens, and less time in LPA and sleep at T2. There was a slight increase in the rate of participation in organized sports clubs from T1 (60%) to T2 (61.5%).

## 4. Discussion

This study examined time spent in PA, SB, screen time, sleep, and associations with executive function of preschool-aged children before and during the COVID-19 pandemic using both repeated cross-sectional and longitudinal data. The repeated cross-sectional data showed that PA and SB levels, screen time, and sleep were similar between pre-pandemic and during-pandemic periods in Japanese preschool-aged children. Even after adjusting for BMI, the differences in PA and SB levels between pre- and during COVID-19 were not statistically significant in the present study, suggesting that COVID-19 has not impacted PA and SB among Japanese preschool children. However, the analysis of longitudinal data showed that the time spent in SB was longer, and the time spent in LPA and sleep were shorter during the pandemic compared to the pre-pandemic period.

A previous systematic review showed that the majority of the COVID-19 studies have used a cross-sectional design, with most studies reporting reduced PA, increased screen time, and longer sleep durations among children and adolescents who live in areas under mild-to-strict lockdown [3]. However, studies conducted in Italy, China, Western Australia, and Poland (where the lockdown restrictions were considered moderate) observed no significant change in PA levels from before to during the lockdown. This is consistent with our findings in the repeated cross-sectional survey, which may be partially explained by the absence of the implementation of lockdown during the pandemic period. However, for organized sports participation, we found a significant difference in the participation rate between the pre-pandemic group (65.1%) and the pandemic group (22.4%). The sports participation rate of the pre-pandemic group was higher by almost 30 percentage points compared with national data from Japan [26], while there was no significant difference in MVPA between the pre- and during-pandemic groups. One of the possible reasons is that accelerometers might have been removed during sports (such as swimming). It could also be that some of the activities performed in the sports club (e.g., how to do breech-over) may not have been reflected in the MVPA estimates. On the other hand, in the longitudinal study, the time spent in LPA was observed to be shorter after participants transitioned from kindergarten to primary school. Previous studies reported that PA levels of first-grade children were lower than those of the final grade preschool children [27,28,29]. Ng et al. [30] reported in their longitudinal analysis that accelerometer-measured MVPA was not negatively impacted by the COVID-19 pandemic among preschoolers in Hong Kong. We suggest that the decline in PA levels (as observed in the longitudinal analysis) may be attributed to the impact of the transition to primary school, which is often accompanied by a number of changes in expectations for learning and behaviors. 

The proportion of participants meeting the SB recommendation was higher in the pre-pandemic group (66.7%) than the pandemic group (57.1%) (Table 2). This is similar to the findings of a previous study involving Japanese children [17]. In the systematic review by Kharel et al. [3], all included studies reported an increase in screen time during the pandemic compared to pre-pandemic period. Using the repeated cross-sectional data, the present study observed no significant differences in SB and screen time from before to during the COVID-19 among preschool children. However, in the longitudinal analysis, the time spent in SB and on screens were found to be longer as children transitioned from kindergarten to primary school during the COVID-19 pandemic. In the previous study, accelerometer-measured SB correlated strongly with LPA for Japanese preschool children (r = −0.95) [31]. On average, an additional 10 min/day of LPA was associated with 12 min/day less of sedentary time. The study indicated that longer daily SB was compensated by shorter LPA. Taylor et al. [29] also reported that screen time of primary school children was longer than that of preschool children. In our systematic review, there was consistent evidence demonstrating that accelerometer-measured SB increases with age among school-age children and adolescents [32]. Jago et al. [33] examined parents’ responses to changes in children’s screen-time between Grade 1 (5–6 years) and Grade 4 (8–9 years of age). Parents reported that the transition from Grade 1 to Grade 4 appeared to be a time during which substantial changes in the screen-based devices that children used and the content that they viewed occurred. Parents also reported that managing screen-viewing was harder as their children got older. In Japan, under the GIGA School Initiative, the deployment of one learning terminal per student in primary and junior high schools nationwide was completed by the end of March 2021 [34]. This Japanese government initiative was accelerated by the COVID-19 pandemic. This may have made screen-based devices easier to use for children in the first grade. Thus, the results of this study did not allow a clear determination of the impact of COVID-19 on children’s screen time.

Most studies included in the systematic review by Kharel et al. [3] reported longer sleep duration during the pandemic compared to the pre-pandemic period among children and adolescents. However, studies conducted in Spain, Australia, the USA, Portugal, and China did not observe significant differences in sleep duration from before to during the lockdown period, which is consistent with our observation in the repeated cross-sectional survey. However, our longitudinal results demonstrated significantly shorter sleep duration as children transitioned to primary school at follow-up, which was attributed to a later bedtime (by 10 min/day) and an earlier wake-up time (by 10 min/day). In other words, the present study found no significant change in bedtime, wake-up time, and quality of sleep both in repeated cross-sectional study and in longitudinal study. Therefore, little change in the children’s life patterns was observed, which may have had almost no effect on sleep habits. However, the proportion of participants meeting the sleep recommendation was slightly higher in the pre-pandemic group (98.4%) than in the pandemic group (91.8%) in the repeated cross-sectional survey, while the proportions were 100% in both T1 and T2 (100%) in the longitudinal study. The proportions in Japanese pre-school children in the previous study were 83.7% in the pre COVID-19 and 79.2% in the pandemic, respectively [17]. The results of the present study did not allow a clear determination of the impact of COVID-19 on children’s sleep time.

Previous reviews reported that young children’s executive functions are associated with PA, SB, and sleep [7,8,9]. As previously discussed, time spent in PA, SB, and sleep were not significantly different between the pre-pandemic group and pandemic group in the repeated cross-sectional study. Moreover, executive functions were not significantly different between the pre-pandemic group and the pandemic group. This finding appears to align with the existing evidence base, as a lack of significant change in PA, SB, and sleep coincided with a lack of significant change in executive functions. 

There are several limitations in the present study. The sample sizes for the repeated cross-sectional and longitudinal analyses were small; therefore, the findings should be interpreted with caution. Furthermore, the longitudinal analysis did not account for the changes in environmental and behavioral factors that accompany the transition from kindergarten to primary school. Therefore, it was not possible to ascertain whether the differences in behaviors are attributed to the impact of COVID-19 or other school transition-related factors. On the other hand, the present participant’s height and body weight were comparable with data in the School Health Survey data by the Ministry of Education, Culture, Sports, Science and Technology [35]. The present study was conducted in public kindergartens and primary schools. Therefore, there may be no substantial bias in the sampling of the target population. Moreover, the lack of executive function data during the follow-up period prevented the ability to conduct a paired-sample analysis to determine if the pandemic affected the executive function of children. However, our study has several strengths. Despite the difficulty in collecting data during the pandemic period, we were able to measure objectively evaluated PA and SB by accelerometers, and executive function was measured with the data collector visiting preschools.

## 5. Conclusions

Children’s PA and SB levels, screen time, and sleep were not significantly different during COVID-19 when compared to data collected in the same pre-schools, using the same methods, prior to the COVID-19 pandemic in Japan. The analysis of longitudinal data demonstrated an increase in time spent in SB and on screens, and a decrease in LPA and sleep during the COVID-19 pandemic than those of the pre-pandemic period. 

Our results suggest that despite of the COVID-19 pandemic, young children’s activity levels and SB have not differed to pre-pandemic levels in Japan. However, school-aged children’s SB, LPA levels, and sleep time have been affected by the COVID-19 pandemic, although a school transition effect cannot be separated. There is an urgent need to understand how long almost no effect on maintained. Trends in SB and screen time among children who experienced the COVID-19 pandemic should be monitored closely in the near future.

## Figures and Tables

**Table 1:** Physical characteristics for participants.

**Cross Sectional Sample**	**Before COVID-19 for Preschool Children (*n* = 63)**			**During COVID-19 for Preschool Children (*n* = 49)**			**Mean Difference**	**95%CI**		***p*-Value**
	**Mean**	**±**	**SD**	**Mean**	**±**	**SD**				
Age (years) (*n* = 63, 49)	6.1	±	0.3	6.1	±	0.3	0.0	−0.1	0.1	1.0
Height (cm)	113.1	±	5.0	113.7	±	5.3	−0.6	−2.6	1.3	0.512
Body weight (kg)	18.5	±	2.4	19.8	±	3.2	−1.3	−2.3	−0.2	0.017
BMI (kg/m^2^)	14.4	±	1.3	15.3	±	1.7	−0.8	−1.4	−0.2	0.006
Weight status (Overweight or obese: %) (*n* = 63, 49)	0%			2.0% (*n* = 1)						
**Longitudinal Sample**	**Before COVID-19 for Preschool Children (*n* = 16)**			**During COVID-19 for Primary School Children (*n* = 16)**			**Mean Difference**	**95%CI**		***p*-Value**
	**Mean**	**±**	**SD**	**Mean**	**±**	**SD**				
Age (years)	6.1	±	0.3	7.2	±	0.3	−1.0	−1.1	−1.0	<0.001
Height (cm)	115.6	±	4.4	119.7	±	4.0	−4.2	−5.5	−2.8	<0.001
Body weight (kg)	19.4	±	1.7	22.0	±	2.8	−2.6	−4.2	−1.0	0.003
BMI (kg/m^2^)	14.5	±	0.9	15.4	±	1.7	−0.8	−2.1	0.4	0.176
Weight status (Overweight and obesity: %)	0%			0%						

BMI: body mass index.

**Table 2:** Proportion of participants meeting the MVPA, screen time, and sleep duration recommendations.

Cross Sectional Sample	Before COVID-19 for Preschool Children (*n* = 63)		During COVID-19 for Preschool Children (*n* = 49)	
	*n*	%	*n*	%
MVPA	57	90.5	45	91.8
Screen time	42	66.7	28	57.1
Sleep	62	98.4	45	91.8
Longitudinal sample	before COVID-19 for preschool children (*n* = 16)		during COVID-19 for primary school children (*n* = 16)	
MVPA	16	100.0	15	93.8
Screen time (*n* = 11)	6	54.5	5	45.5
Sleep (*n* = 13)	13	100.0	13	100.0

MVPA: moderate- to vigorous-intensity physical activity.

**Table 3:** Comparison of time spent in sedentary behavior, physical activity, sleep, and executive function before and during COVID-19.

	Before COVID-19 for Preschool Children (*n* = 63)			During COVID-19 for Preschool Children (*n* = 49)			B	95%CI		*p*-Value
	Mean	±	SD	Mean	±	SD	Adjusted for Sex, School and Wearing Time			
Sedentary behavior (min/day)	586.5	±	52.1	605.4	±	49.3	−6.302	−22.8	10.2	0.450
LPA (min/day)	103.3	±	16.8	97.5	±	18.7	6.395	−0.1	12.9	0.055
MVPA (min/day)	102.2	±	32.3	99.7	±	32.4	−0.093	−11.7	11.5	0.987
VPA (min/day)	24.0	±	14.8	26.9	±	12.4	−3.808	−9.1	1.5	0.154
							Adjusted for sex and school			
TV time (min/day)	85.1	±	62.6	87.1	±	74.5	−9.173	−36.4	18.0	0.505
Game time (min/day)	36.4	±	52.6	57.2	±	86.6	−22.254	−50.0	5.5	0.115
Screen time (min/day)	121.5	±	83.8	144.3	±	128.5	−31.427	−73.8	11.0	0.145
Sleep duration (min/day)	607.6	±	28.8	608.6	±	33.5	−1.517	−13.8	10.8	0.807
Bedtime (h:min)	21:03	±	:33	21:04	±	:46	−3.267	−19.2	12.7	0.685
Wake-up time (h:min)	7:11	±	:36	7:11	±	:40	−2.504	−17.9	12.9	0.748
Inhibition (score)	0.77	±	0.18	0.78	±	0.16	0.004	0.9	0.1	0.913
Working memory (score) (*n* = 63, 47)	2.83	±	0.98	2.75	±	1.14	−0.026	−0.5	0.4	0.906
Cognitive flexibility (score)	9.10	±	3.03	9.18	±	1.92	−0.225	−1.3	0.8	0.671

LPA: Light-intensity physical activity, MVPA: moderate- to vigorous-intensity physical activity, VPA: vigorous-intensity physical activity.

**Table 4:** Change of habitual sedentary behavior, physical activity, and sleep for participants baseline and follow-up.

	Before COVID-19			During COVID-19 for Primary School Children			Mean Difference	95%CI		*p*-Value
	Mean	±	SD	Mean	±	SD				
Sedentary behavior (min/day) (*n* = 16)	576.4	±	48.2	613.6	±	51.5	−37.2	−62.0	−12.3	0.006
LPA (min/day) (*n* = 16)	110.0	±	15.0	97.5	±	19.1	12.5	3.4	21.6	0.011
MVPA (min/day) (*n* = 16)	119.2	±	31.4	106.9	±	32.8	12.3	−1.9	26.4	0.085
VPA (min/day) (*n* = 16)	28.8	±	17.1	31.1	±	14.8	−2.3	−14.7	10.1	0.693
TV time (min/day) (*n* = 11)	67.8	±	25.2	104.0	±	52.0	−36.2	−80.3	7.8	0.097
Game time (min/day) (*n* = 11)	36.8	±	46.8	64.7	±	74.7	−27.9	−58.2	2.4	0.067
Screen time (min/day) (*n* = 11)	104.6	±	54.3	168.7	±	105.7	−64.1	−116.8	−11.5	0.022
Sleep duration (min/day) (*n* = 13)	603.1	±	26.3	583.3	±	26.3	19.8	2.7	36.8	0.027
Bedtime (h:min) (*n* = 13)	21:01	±	:23	21:10	±	:40	−8.8	−31.1	13.4	0.404
Wake-up time (h:min) (*n* = 13)	7:04	±	:26	6:53	±	:29	10.8	−12.7	34.3	0.335

LPA: Light-intensity physical activity, MVPA: moderate- to vigorous-intensity physical activity, VPA: vigorous-intensity physical activity.

## Data Availability

The data that support the findings of this study are available from the corresponding authors upon reasonable request.
